# Severe abacavir hypersensitivity reaction in a patient with human immunodeficiency virus infection: a case report

**DOI:** 10.1186/s13256-022-03647-6

**Published:** 2022-11-08

**Authors:** Mathew K. Koech, Shamim M. Ali, Mercy J. Karoney, Gabriel Kigen

**Affiliations:** 1 Department of Medicine, School of Medicine, College of Health Sciences, Moi University, Eldoret, Kenya; 2 Moi Teaching and Referral Hospital, Eldoret, Kenya; 3 Department of Pharmacology & Toxicology, School of Medicine, College of Health Sciences, Moi University, Eldoret, Kenya

**Keywords:** Abacavir, Abacavir hypersensitivity, ABC HSR, HLA-B*57:01, Antiretroviral, HIV

## Abstract

**Background:**

Abacavir is a nucleoside reverse transcriptase inhibitor that is used as a component of the antiretroviral treatment regimen in the management of the human immunodeficiency virus for both adults and children. It is efficacious, but its use may be limited by a hypersensitivity reaction linked with the HLA-B*57:01 genotype. HLA-B*57:01 has been reported to be rare in African populations. Because of the nature of its presentation, abacavir hypersensitivity is prone to late diagnosis and treatment, especially in settings where HLA-B*57:01 genotyping is not routinely done.

**Case report:**

We report a case of a severe hypersensitivity reaction in a 44-year-old Kenyan female living with the human immunodeficiency virus and on abacavir-containing antiretroviral therapy. The patient presented to the hospital after recurrent treatment for a throat infection with complaints of fever, headache, throat ache, vomiting, and a generalized rash. Laboratory results evidenced raised aminotransferases, for which she was advised to stop the antiretrovirals that she had recently been started on. The regimen consisted of abacavir, lamivudine, and dolutegravir. She responded well to treatment but was readmitted a day after discharge with vomiting, severe abdominal pains, diarrhea, and hypotension. Her symptoms disappeared upon admission, but she was readmitted again a few hours after discharge in a hysterical state with burning chest pain and chills. Suspecting abacavir hypersensitivity, upon interrogation she reported that she had taken the abacavir-containing antiretrovirals shortly before she was taken ill. A sample for HLA-B*57:01 was taken and tested positive. Her antiretroviral regimen was substituted to tenofovir, lamivudine, and dolutegravir, and on subsequent follow-up she has been well.

**Conclusions:**

Clinicians should always be cognizant of this adverse reaction whenever they initiate an abacavir-containing therapy. We would recommend that studies be done in our setting to verify the prevalence of HLA-B*57:01.

**Supplementary Information:**

The online version contains supplementary material available at https://doi.org/10.1186/s13256-022-03647-6.

## Background

Abacavir (ABC), is a nucleoside reverse transcriptase inhibitor that is used as a component of the antiretroviral treatment (ART) regimen in the management of the human immunodeficiency virus (HIV) for both adults and children [1, 2]. Current ABC-containing fixed-dose regimens include its co-formulation with lamivudine (3TC) alone, or in combination with either zidovudine (AZT) or dolutegravir (DTG) [3, 4]. Despite its efficacy, ABC has been associated with several adverse effects including hepatotoxicity, lactic acidosis, and a hypersensitivity reaction commonly referred to as abacavir hypersensitivity reaction (ABC HSR) [5–7]. ABC HSR has been linked to the HLA-B*57:01 gene, and studies indicate that 5–8% of the population are at risk of developing this adverse reaction, which may be severe to potentially life threatening [8, 9]. However, studies conducted in Europe have reported a higher prevalence in Caucasian (6.5%) compared with African communities (0.4%) [10].

This reaction occurs within the first 6 weeks of ABC therapy, with a median onset of 11 days [8, 11–14]. The adverse effects manifest with multiple constitutional symptoms including fever, maculopapular rash, myalgia, chills, headache, chronic pain, fatigue, dyspnea, and malaise. It may also present with gastrointestinal and/or respiratory symptoms [12, 13, 15, 16]. The symptoms have been reported to occur suddenly, and progressively with each consecutive dose [14, 17]. This array of symptoms delays the diagnosis and subsequent treatment, more so in resource-limited settings where testing for HLA-B*57:01 is not routinely carried out before ABC is started. Confirmatory diagnosis is through a genetic test for the HLA-B*57:01 allele, which may also be used to predict the adverse event [18–20]. Management involves immediate discontinuation, and fatality may result upon reintroduction [16, 21, 22]. A rechallenge with ABC is therefore absolutely contraindicated [12, 23, 24]. Improvement, however, occurs within 48–72 hours of withdrawal, although complete resolution may take longer [14, 25, 26].

In Kenya, the backbone of antiretroviral treatment remains tenofovir (TDF) [27]. With increasing concerns for the adverse effects of TDF, there has been an increase in the use of ABC. This poses a challenge given the ABC HSR and the lack of routine screening for HLA-B*57:01.

We report a case of ABC HSR in a patient on an ABC-containing ART regimen for the treatment of human immunodeficiency virus (HIV) infection, which resolved upon withdrawal of the drug.

## Case presentation

A 44-year-old female living with HIV and on ART for the last 14 years, with a CD4 count of 338 cells/µL and viral load < 20 copies/mL presented to the hospital with a history of being unwell for a week. She had been put on abacavir, lamivudine, and dolutegravir 3 weeks before, as a routine switch from a TDF-based regimen. She had previously been on a regimen of TDF, 3TC, and efavirenz but had it changed because of the availability of a new formulation of ABC, 3TC, and DTG (Triumeq) that was deemed better by her primary physician. She reported a dry cough, sore throat, chest discomfort, and myalgia. She also reported a history of vomiting 3 days before the hospital visit. She was subject to fever and chills, was anorexic and nauseous. She also complained of occasional headaches, without neck stiffness or photophobia. She had been tested for malaria a couple of times a few days prior, but was persistently negative. At presentation, her blood pressure was 142/84 mmHg, heart rate 102 beats per minute, with temperature 36.1 °C and oxygen saturation at 94% on ambient air.

On examination, she had an inflamed throat and posterior palate with tender tonsils to palpation, her chest was clear and the cardiovascular system examination was otherwise unremarkable. She also had tenderness to palpation of the limb muscles without evidence of arthritis. Remarkably, she had a white cell count of 2.6 × 10^9^/L (73% granulocytes, 22% lymphocytes), hemoglobin of 14.0 g/dL, and platelet count of 122 × 10^9^/L, but her kidney function tests were within normal. The rest of the laboratory results are outlined in Additional file 1: Table S1. She was admitted for intravenous rehydration and bed rest, with a presumption of a viral upper respiratory tract infection. However, the sore throat persisted despite the initial relief. On the second day of admission, she complained of generalized body ache. Laboratory results showed a significant increase in liver enzymes, thus prompting testing for viral hepatitis and repeat testing for malaria, which turned positive, and she was subsequently started on artesunate. The patient was advised to suspend her ART given the liver impairments. She responded well to treatment and the transaminases started to improve, although she kept complaining of myalgias and progressive body swelling with paresthesia in the lower limbs. She was discharged on the seventh day feeling better.

She was, however, readmitted a day after discharge (ninth day since the first admission) complaining of an episode of severe headache followed by profuse sweating. She denied fever but reported that she had vomited six times that day and had three episodes of diarrhea with burning lower abdominal pains. Her blood pressure was 87/50 mmHg, heart rate 130 beats per minute, temperature 35.4 °C, and oxygen saturation at 88%. Apart from a tender epigastrium, systemic examination was otherwise unremarkable. She was started on rabeprazole, antibiotics to cover hospital acquired infection, and ondansetron. Unfortunately, cultures were not taken. She was also started on Ringer’s lactate. Significantly, her laboratory results showed features of acute kidney injury (AKI) with a creatinine of 183 µmol/L. On the second day of this admission, she developed a rash in the extremities that spread to the trunk, she was started on cetirizine and mometasone cream. As her blood pressure improved, the kidney function improved. In addition, there was a notable improvement in the liver transaminases compared with previous tests. The rash also improved. She was discharged after 5 days (13 days after the initial admission).

However, the patient was readmitted the night of the day of discharge, in a hysterical state with burning chest pain and chills. Her blood pressure was 108/67 mmHg, heart rate 153 beats per minute, temperature 34.2 °C, and oxygen saturation at 92% on room air. Systemic examination was unremarkable. There was no notable rash at this time. A bedside electrocardiogram was unremarkable apart from the extreme tachycardia and an arterial blood gas report consistent with respiratory alkalosis with lactate of 4.3 mmol/L. However, there was a highly elevated serum level of aspartate aminotransferase, lactate dehydrogenase, and potassium. On further interrogation, the patient revealed that she had taken the ABC-containing tablets shortly before she became unwell and she had vomited a while after that. It was also noted that the patient had been restarting her ART every time she was discharged, contrary to advice.

**Fig. 1:**
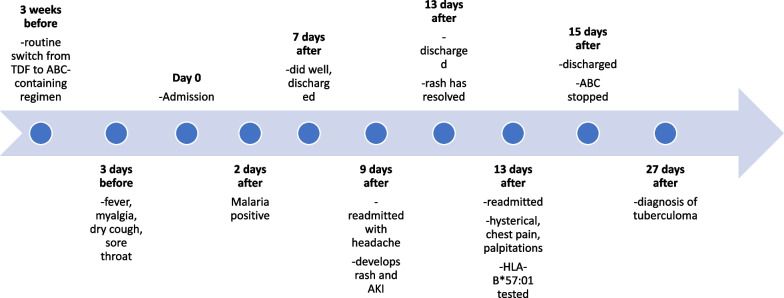
Timeline of events

The patient was managed supportively and showed marked improvement, although she still had peripheral neuropathy of the left lower limb requiring physiotherapy and the use of a crutch. On clinical suspicion of abacavir hypersensitivity, an HLA-B*57:01 test was requested. This proved positive and the patient was advised to completely stop the ABC/DTG/3TC regimen, as she was hypersensitive to ABC. She was discharged after a 2-day stay at the hospital. On follow-up, she did well. She presented 12 days later (27 days after the initial presentation) with severe headaches and episodes of confusion. A brain computed tomography (CT) scan and magnetic resonance imaging (MRI) scan revealed a left posterior parietal tuberculoma. She was started on rifampicin, isoniazid, pyrazinamide, and ethambutol for 2 months, and then rifampicin and isoniazid for 16 months (this was occasioned by persistent symptoms at 12 months and persistent but shrinking tuberculoma). She received a short course of dexamethasone at the beginning of this treatment. Two months after the initial presentation, her ART regimen was changed to a two-tablet regime consisting of DTG and a fixed-dose combination of emtricitabine plus TDF. This was occasioned by a rise in the HIV viral load with stable liver and kidney function test results while on antituberculous therapy. She made tremendous improvement overall, although she has persistent headaches 24 months after the first episode. The most recent CD4 count is 512 cells/µL and viral load is 22 copies/mL. The timeline of events is summarized in Fig. 1.

## Discussion

This report highlights the challenges involved in making a diagnosis of ABC HSR, especially in resource-limited settings. Our patient exacerbated the situation by reintroducing the ABC-containing regimen by herself. The coexistence of malaria early on contributed to the symptomatology and could have explained the deranged liver functions, further delaying the diagnosis. It is possible that some of the symptoms she presented with earlier, especially the intermittent headaches and vomiting, could have been attributed to the diagnosis of tuberculoma that only became apparent later on.

ABC HSR is rare in people originating from sub-Saharan Africa, with a reported overall prevalence of 0.1–0.8% in Kenya [28, 29]. It is a significant idiosyncratic adverse reaction that is complicated by a wide spectrum of clinical symptoms, without marked skin-associated manifestations as widely believed. It may be life threatening, especially on rechallenge [11, 17, 22, 24]. The variable onset to development of clinical symptoms, and the recognition that the hypersensitivity in HIV may result from other co-administered drugs further complicates the diagnosis [5, 30, 31]. The ideal situation would be routine screening of all patients for HLA-B*57:01 before the introduction of abacavir-containing regimens. Unfortunately, this is not routinely recommended in resource-limited settings. Epicutaneous patch testing may be used [8, 18–20, 32, 33]. However, this is not feasible in resource-limited settings, which coincidentally bear the brunt of the disease.

Moreover, the known epidemiology of ABC HSR makes it difficult to justify routine testing for HLA-B*57:01 in our setting. HLA-B*57:01 positivity is more prevalent in Caucasian populations and is significantly less in African populations [34].

The mechanisms for ABC HSR are incompletely understood. It is thought to result from conformational changes arising from the binding of ABC to HLA-B*5701 peptides [35]. Abacavir binds with high specificity to the HLA-B*5701 peptides, thereby altering the shape of the antigen-binding cleft, resulting in a change of immunological tolerance and activation of abacavir-specific cytotoxic T cells, which trigger a hypersensitivity reaction [35, 36].

Once a diagnosis of ABC HSR is made, it is very important to instruct the patient to never take ABC as it is potentially fatal to do so. The patient in this case kept taking the ABC, rightfully worried about adherence to her HIV medication, but once the test result confirmed ABC HSR she got a clear instruction from the team.

To the best of our knowledge, this case report is the first ever reported case of ABC HSR from Kenya and the region. Researchers have however reported a 0.8% prevalence of the HLA-B*57:01 allele in the Kenyan population, based on results from screening of samples from HIV-positive patients [29].

## Conclusions

We have presented a case of ABC HSR that was a diagnostic challenge because it was initially not thought of and the patient kept restarting her ART without informing the treating physicians. Clinicians should always therefore be cognizant of this adverse reaction whenever they initiate ABC-containing therapy. We would recommend that studies be done in our setting to verify the prevalence of HLA-B*57:01.

## Supplementary Information

**Additional file 1. ** Lab data.

## Data Availability

All available data are included in Additional file 1: Table S1.

## Abbreviations

3TC: Lamivudine
ABC: Abacavir
ABC HSR: Abacavir hypersensitivity reaction
ART: Antiretroviral treatment
AZT: Zidovudine
CT: Computed tomography
DTG: Dolutegravir
HIV: Human immunodeficiency virus
MRI: Magnetic resonance imaging
